# Prognostic impact of visceral and subcutaneous fat area in stage I-III colon cancer patients with cachexia: a population-based multicenter study

**DOI:** 10.3389/fnut.2025.1538285

**Published:** 2025-03-03

**Authors:** Xian-wen Liang, Jing Wen, Bing Liu, Sheng-zhong Wang, Jin-cai Wu, Tao Pan

**Affiliations:** ^1^Department of Gastrointestinal Surgery, Hainan General Hospital (Hainan Affiliated Hospital of Hainan Medical University), Haikou, China; ^2^Department of Gastrointestinal Surgery, Central South University Xiangya School of Medicine Affiliated Haikou Hospital, Haikou, China; ^3^Department of Gastrointestinal Surgery, Chengdu Second People's Hospital, Chengdu, China; ^4^Department of Colorectal Cancer Surgery, Sichuan Clinical Research Center for Cancer, Sichuan Cancer Hospital & Institute, Sichuan Cancer Center, Affiliated Cancer Hospital of University of Electronic Science and Technology of China, Chengdu, China

**Keywords:** colon cancer, cancer cachexia, visceral fat area, subcutaneous fat area, overall survival

## Abstract

**Background:**

Adipose tissue reduction is one of the features in patients with cancer cachexia. However, it remains unclear whether visceral fat area (VFA) and subcutaneous fat area (SFA) contribute differently to the progression of cancer cachexia in colon cancer patients. This study aims to investigate the prognostic impact of VFA and SFA in stage I-III colon cancer patients with cachexia.

**Methods:**

Patients diagnosed with stage I-III colon cancer were preoperatively measured for VFA and SFA and then divided into VFA-high (VFA-H) and VFA-low (VFA-L) groups, as well as SFA-high (SFA-H) and SFA-low (SFA-L) groups. The prognostic impact of VFA and SFA for colon cancer patients with cachexia were analyzed using the Kaplan–Meier method and Cox regression analysis.

**Results:**

A total of 916 colon cancer patients (377 with cachexia and 539 without) were included in the study. In patients with cachexia, the estimated five-year overall survival (OS) was higher in the VFA-H group compared to the VFA-L group (*p* < 0.001). There was no significant difference in five-year OS between the SFA-L and SFA-H groups (*p* = 0.076). Cox regression analysis indicated that VFA (hazard ratio [HR] = 0.55, 95% confidence interval [CI] 0.40 to 0.76; *p* < 0.001) was an independent prognostic factor for patients with cachexia. SFA (HR = 0.78, 95% CI 0.59 to 1.03; *p* = 0.076) was not an independent prognostic factor for patients with cachexia.

**Conclusion:**

Preoperative VFA, but not SFA was a useful prognostic factor for long-term outcomes in stage I-III colon cancer patients with cachexia. More attention should be paid to VFA in colon cancer patients with cachexia.

## Introduction

According to the global cancer data of 2023, colorectal cancer (CRC) ranks third in both incidence and mortality among all malignant tumors (1). The incidence of cancer cachexia varies across various cancer types, with its prevalence in CRC approaching approximately 50% (2). Cancer cachexia represents a high metabolic state in cancer patients, characterized by significant muscle atrophy and the loss of adipose tissue, conditions not fully reversed through standard nutritional support (3). The progression of cancer cachexia significantly decreases both the efficacy of therapeutic interventions and overall survival, and is identified as the direct cause of death in 25% of cancer patients (2). However, cancer cachexia is often underrecognized and inadequately treated (4, 5). Although previous studies have primarily focused on the effects of muscle atrophy in this condition (2, 6), emerging evidence suggests that the loss of adipose tissue also plays a significant role in the progression of cancer cachexia (7).

Adipose tissue, characterized by its functional and histological heterogeneity, is distributed throughout the body. White adipose tissue, functioning primarily as an energy reservoir, is anatomically categorized into visceral adipose tissue and subcutaneous adipose tissue (8). These adipose tissue types are marked by their unique variations, with differences extending across anatomical, cellular, molecular, physiological, clinical, and prognostic aspects (9). Adipose tissue reduction also contributes to the devastating impact of cancer cachexia, manifested by increased energy expenditure, decreased quality of life, and shortened survival duration (10, 11).

However, the effect of adipose tissue reduction, specifically subcutaneous fat area (SFA) and visceral fat area (VFA), on the prognosis of cancer patients with cachexia remains unknown. In this study, we conducted a comparative analysis of SFA, VFA, and body mass index (BMI) in colon cancer patients with cachexia to assess their respective prognostic implications on the outcome of these patients.

## Methods

### Ethical issue

This study was conducted with the approval of the ethics committees of Haikou Hospital and the ethics committees of Chengdu Second People’s Hospital and adhered to the STROBE guidelines for observational studies (12). Due to the retrospective design, the patient data were anonymized prior to analysis, and the requirement for individual informed consent was exempted.

### Study population

Between January 2013 and December 2018, a total of 916 patients underwent radical colectomy for colon cancer at Central South University Xiangya School of Medicine Affiliated Haikou Hospital (Haikou Hospital), and Chengdu Second People’s Hospital were included in the study (Supplementary Figure S1). These cases were identified from prospective databases. Participants were included based on the following criteria: (1) age ≥ 18 years; (2) histological confirmation of colon cancer through biopsy; (3) underwent radical colectomy; and (4) with abdominal CT scans conducted within 1 month before surgery and measurements of VFA and SFA available. The exclusion criteria were: (1) with any preoperative oncologic treatments; (2) palliative surgery; (3) synchronous colorectal carcinoma; (4) incomplete medical records; and (5) loss to follow-up.

Each patient was followed up periodically through outpatient visits, phone interviews, and in hospital settings, every 3 months in the first 2 years, every 6 months during the third to fifth years, and annually after 5 years. The study also recorded patients who were lost to long-term follow-up, mainly due to their inability to return for outpatient visits or issues with their contact information that prevented further contact. As of December 2023, out of 992 patients, 916 (92.3%) had complete follow-up data. The median follow-up period was 73 months. The primary outcome of the study was overall survival (OS) after surgery.

### Clinicopathological characteristics and definition

The clinicopathologic data reviewed included preoperative parameters: age, sex, BMI (kg/m^2^), American Society of Anesthesiology (ASA) score, pre-existing co-morbidities (including heart disease, hypertension requiring medication, chronic pulmonary disease, diabetes mellitus), preoperative albumin (g/L), CEA level (ng/ml), tumor location (right-sided, left-sided), SFA (cm^2^), and VFA (cm^2^). Operative parameters included surgical approach (laparoscopy, laparotomy). Postoperative parameters included pathological TNM stage (pTNM), lymphovascular invasion, neural invasion, and tumor differentiation.

The TNM stage was determined according to the eighth edition of the American Joint Committee on Cancer (AJCC) Staging Manual (13). Right-and left-sided colon cancer were defined as proximal and distal to the splenic flexure, respectively, (14). Cancer cachexia was diagnosed based on the 2011 Fearon Consensus (3). A diagnosis of cancer cachexia can be made if the patient meets any of the following criteria: (1) unintentional weight loss exceeding 5% in the past 6 months; (2) BMI lower than 20 kg/m^2^ accompanied by any degree of unintentional weight loss exceeding 2% in the past 6 months; (3) the presence of sarcopenia along with any degree of unintentional weight loss exceeding 2% in the past 6 months.

### Fat area measurement and cut-off point

**Figure 1:**
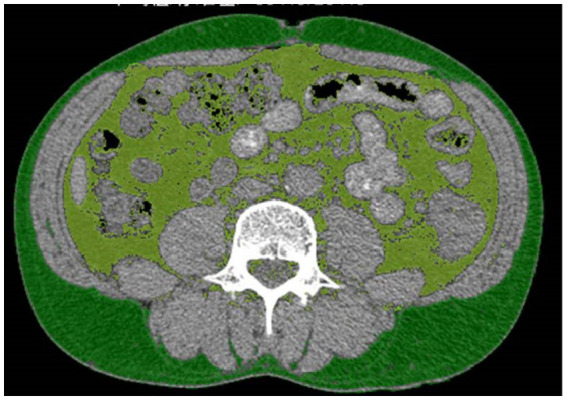
The measurement of the visceral fat area (VFA) and subcutaneous fat area (SFA). Light green represents VFA, dark green represents SFA.

All patients in the study received abdominal computed tomography (CT) scans as a preoperative evaluation. SFA and VFA were measured using a cross-sectional image from CT scan of the abdomen at the level of umbilicus (15, 16). On CT scans, the identification of adipose tissue was achieved by setting the attenuation values within the range of −190 to −30 Hounsfield units (HU) (17, 18). The contour of the subcutaneous fat tissue and the outline of the visceral fat tissue were obtained by a fat assessment tool (19) (Siemens Healthineers Syngo via Frontier, USA), as shown in Figure 1. The measurements were conducted by a radiologist blinded to patient information. These data acquisitions were all achieved within Siemens Healthineers Syngo software application.

Patients were categorized into two groups based on their VFA, SFA, and BMI. Patients were classified into a VFA-L group (VFA < 100 cm^2^) and a VFA-H group (VFA ≥ 100 cm^2^) using 100 cm^2^ as the cut-off value for VFA according to the Japan Society for the Study of Obesity (20). Several earlier studies have also adopted this cut-off point (18, 21–23). To the best of our knowledge, there has been no classification based on SFA values. Following a methodology from a previous study (16), SFA were dichotomised using the median as the cut-off point, resulting in an SFA-L group (SFA < the median) and an SFA-H group (SFA ≥ the median). BMI were dichotomised using the Asian-Pacific standard (24), resulting in a BMI-L group (BMI < 25 kg/m^2^) and a BMI-H group (SFA ≥ 25 kg/m^2^).

### Statistical methods

The normality tests of the data were performed routinely before statistical analysis by parametric tests. Continuous variables were represented by median and interquartile range (IQR) and categorical variables were expressed as number and percentage. Continuous data were compared with independent sample t test or Mann–Whitney U test, and categorical data were compared with Fisher’s exact test or Pearson χ2 test. OS was calculated according to Kaplan–Meier method and compared by the log-rank test. The survival curves were constructed by the packages of “ggplot2,” “survminer,” and “survival” in R. The correlation analysis was constructed by the packages of “ggplot2” in R. The Cox proportional hazard regression model with conditional backward stepwise analysis was conducted to perform univariate and multivariate survival analysis. Statistical analysis was performed using SPSS 19.0 (SPSS®, Chicago, IL, USA) and R (Version 4.3.2).^1^ A two-tailed *p* < 0.05 was considered statistically significant.

## Results

### Baseline characteristics

This study included 916 colon cancer patients. The baseline characteristics are detailed in Supplementary Table S1. Patients were classified into non-cachexia (*n* = 539) and cachexia (*n* = 377) groups. The comparisons of characteristics between these two groups are shown in Table 1. Cachexia was significantly associated with older age, higher ASA score, elevated CEA levels, decreased albumin, chronic pulmonary disease, diabetes mellitus, BMI, reduced VFA and SFA, and higher tumor, node, and TNM stages.

**Table 1:** Characteristics of colon cancer patients with and without cachexia.

Clinicopathologic characteristics	Non-cachexia	Cachexia	*p*†
	*n* = 539	*n* = 377	
Sex			0.109
Male	306 (56.8)	234 (62.1)	
Female	233 (43.2)	143 (37.9)	
Age (year)*	60.0 (50.5, 68.0)	61.0 (54.0, 69.0)	0.003‡
ASA score			<0.001
1–2	439 (81.4)	151 (40.1)	
3–4	100 (18.6)	226 (59.9)	
Hypertension			0.996
No	449 (83.3)	314 (83.3)	
Yes	90 (16.7)	63 (16.7)	
Heart disease			0.659
No	522 (96.8)	367 (97.3)	
Yes	17 (3.2)	10 (2.7)	
Chronic pulmonary disease			0.031
No	509 (94.4)	342 (90.7)	
Yes	30 (5.6)	35 (9.3)	
Diabetes mellitus			0.010
No	497 (92.2)	328 (87.0)	
Yes	42 (7.8)	49 (13.0)	
CEA level (ng/ml)*	2.4 (1.4, 4.8)	3.0 (1.7, 9.2)	<0.001‡
Albumin (g/L)*	40.1 (37.9, 42.0)	34.9 (32.7, 37.7)	<0.001‡
BMI (kg/m^2^)*	22.8 (21.1, 24.7)	21.9 (19.6, 23.5)	<0.001‡
VFA (cm^2^)*	88.9 (66.6, 125.0)	64.5 (33.9, 104.6)	<0.001‡
SFA (cm^2^)*	116.3 (80.7, 158.9)	88.3 (61.3, 121.0)	<0.001‡
Surgical approach			0.883
Laparoscopy	342 (63.5)	241 (63.9)	
Laparotomy	197 (36.5)	136 (36.1)	
Pathologic tumor category			<0.001
T1-2	74 (13.7)	18 (4.8)	
T3-4	465 (86.3)	359 (95.2)	
Pathologic node category			<0.001
N0	380 (70.5)	187 (49.6)	
N1-2	159 (29.5)	190 (50.4)	
AJCC 8th staging			<0.001
I	64 (11.9)	7 (1.9)	
II	316 (58.6)	180 (47.7)	
III	159 (29.5)	190 (50.4)	
Perineural invasion			0.088
No	465 (86.3)	305 (80.9)	
Yes	62 (11.5)	59 (15.6)	
Not reported	12 (2.2)	13 (3.4)	
Lympho-vascular invasion			0.375
No	436 (80.9)	291 (77.2)	
Yes	97 (18.0)	80 (21.2)	
Not reported	6 (1.1)	6 (1.6)	
Tumor differentiation			0.111
Well	167 (31.0)	93 (24.7)	
Moderate	309 (57.3)	234 (62.1)	
Poor	63 (11.7)	50 (13.3)	
Adjuvant chemotherapy			0.834
No	304 (56.4)	210 (55.7)	
Yes	235 (43.6)	167 (44.3)	

Values in parentheses are percentages unless indicated otherwise; *values are median (interquartile range). ^†^χ2 or Fisher’s exact test, except ‡Student’s t test. ASA, American Society of Anesthesiology; BMI, body-mass index; VFA, visceral fat area; SFA, subcutaneous fat area.

### Fat parameters and their correlations

As shown in Supplementary Table S1, median BMI was 22.4 kg/m^2^ (IQR, 20.4–24.3), VFA was 83.1 cm^2^ (IQR, 52.8–114.8), and SFA was 103.6 cm^2^ (IQR, 72.9–152.1), establishing an SFA cutoff value at 103.6 cm^2^. Correlation analysis showed significant correlations between BMI and VFA (*r* = 0.615, *p* < 0.001), BMI and SFA (*r* = 0.661; *p* < 0.001), and SFA and VFA (*r* = 0.552; *p* < 0.001).

### Survival analysis of patients with cachexia

**Figure 2:**
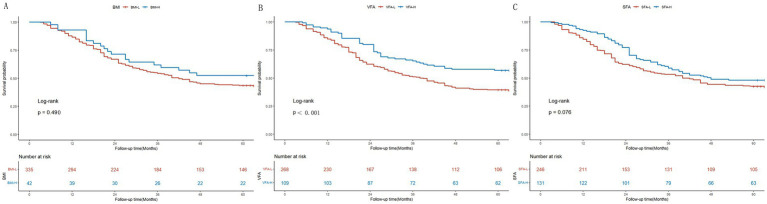
Kaplan–Meier survival curves for colon cancer patients with cachexia. **(A)** Stratification by BMI (BMI-L vs. BMI-H); **(B)** Stratification by VFA (VFA-L vs. VFA-H); **(C)** Stratification by SFA (SFA-L vs. SFA-H).

As depicted in Supplementary Figure S2, the estimated five-year OS was significantly higher in the non-cachaxia group compared with the cachexia group in the present study (*p*<0.001; Supplementary Figure S2). Patients with cachexia were categorized based on their BMI, VFA, and SFA. As depicted in Figure 2, the five-year OS showed no significant difference between the BMI-L and BMI-H groups (*p* = 0.210; Figure 2A). Conversely, the estimated five-year OS was higher in the VFA-L group compared to the VFA-H group (*p*<0.001; Figure 2B). The five-year OS showed no significant difference between the SFA-L and SFA-H groups (*p* = 0.076; Figure 2C).

### Cox multivariate survival analysis

As shown in Table 2, the Cox univariate and multivariate analysis indicated that TNM stage (hazard ratio [HR] = 1.60, 95% confidence interval [CI] 1.23 to 2.21; *p* = 0.001), and VFA (HR = 0.55, 95% CI 0.40 to 0.76; *p* < 0.001) were independent prognostic factors for colon cancer patients with cachaxia. SFA (HR = 0.78, 95% CI 0.59 to 1.03; *p* = 0.076) was not independent prognostic factor for colon cancer patients with cachaxia.

**Table 2:** Univariate and multivariate survival analysis of patient with cachexia by Cox proportion hazard model.

	Univariate analysis		Multivariate analysis	
	Hazard ratio	*p*	Hazard ratio	*p*
Sex		0.332		
Male	1.00 (reference)			
Female	0.88 (0.67, 1.15)			
Age (years)		0.066		
<60	1.00 (reference)			
≥60	1.28 (0.98, 1.67)			
Hypertension		0.561		
No	1.00 (reference)			
Yes	1.02 (0.61, 1.56)			
Heart disease		0.123		
No	1.00 (reference)			
Yes	1.11 (0.81, 1.39)			
Chronic pulmonary disease		0.061		
No	1.00 (reference)			
Yes	1.31 (0.97, 1.41)			
Diabetes mellitus		0.056		
No	1.00 (reference)			
Yes	1.39 (0.99, 1.51)			
BMI (kg/m^2^)		0.494		
<25	1.00 (reference)			
≥ 25	0.87 (0.57, 1.31)			
VFA		<0.001		<0.001
VFA-L	1.00 (reference)		1.00 (reference)	
VFA-H	0.57 (0.42,0.78)		0.55 (0.40, 0.76)	
SFA		0.076		
SFA-L	1.00 (reference)			
SFA-H	0.78 (0.59, 1.03)			
AJCC 8th staging		<0.001		0.001
I + II	1.00 (reference)		1.00 (reference)	
III	1.61 (1.24, 2.10)		1.60 (1.23, 2.21)	
Perineural invasion		0.042		0.226
No	1.00 (reference)		1.00 (reference)	
Yes + Not reported	1.40 (1.01, 1.92)		1.23 (0.88, 1.70)	
Lympho-vascular invasion		0.072		
No	1.00 (reference)			
Yes + Not reported	1.31 (0.98, 1.77)			
Tumor differentiation				
Well	1.00 (reference)			
Moderate + Poor	1.05 (0.70, 1.57)			
Adjuvant chemotherapy		0.666		
No	1.00 (reference)			
Yes	0.94 (0.73, 1.23)			

Values in parentheses are 95% confidence intervals. BMI, body-mass index; VFA, visceral fat area; SFA, subcutaneous fat area.

## Discussion

Previous studies have highlighted the limitations of using BMI as a prognostic indicator in cancer patients (25–27). The discrepancies observed in these studies can be attributed to BMI’s inability to accurately reflect an individual’s body composition. In this study, we explore the relationship between body composition, specifically VFA and SFA, and the survival of colon cancer patients. This study indicated that preoperative VFA was found to be a useful prognostic factor for long-term outcomes in stage I-III colon cancer with cachexia. Preoperative CT measurement of VFA could aid in identifying and stratifying patients with poor prognoses, which might be beneficial for improving the prognosis in colon cancer patients with cachexia. To the best of our knowledge, this was the first study to investigate the prognostic value of VFA and SFA in colon cancer patients with cachexia.

Previous studies on the prognostic impact of VFA in colorectal cancer patients have yielded divergent outcomes (28–31). However, approximate 50% of colon cancer patients may develop cachexia (2), and cancer cachexia is often underrecognized and inadequately treated (4). The estimated five-year OS was significantly higher in the non-cachaxia group compared with the cachexia group in the present study (Supplementary Figure S2). Therefore, the identification of colon cancer patients with cachexia becomes particularly important. Identifying prognostic factors related to these patients is of great value in guiding their treatment.

Previous studies have suggested that visceral adipose tissue and subcutaneous adipose tissue have distinct characteristics and functional roles in metabolic regulation (9, 32). There are likely intrinsic differences between them. Previous studies have also shown that higher VFA is associated with an increased risk of postoperative complications in various cancers (18, 33, 34). In this study, we found that colon cancer patients with cachexia had lower VFA and SFA compared with those without cachexia. We also found that VFA, rather than SFA, emerged as an independent prognostic indicator for colon cancer patients with cachexia. Our results are in contrast to another study (23), which found that the prognosis of gastric cancer patients with cachexia was related to SFA. This might attribute to the different cancer types.

The reason why VFA, rather than SFA, emerged as a significant prognostic factor in our study may be explained by the unique biological and metabolic roles of visceral fat. VFA is more metabolically active than SFA and plays a central role in inflammation, metabolic dysregulation, and energy homeostasis (9). In particular, VFA is a key source of pro-inflammatory cytokines and adipokines, which are closely associated with the development and progression of cancer cachexia. The accumulation of visceral fat can exacerbate systemic inflammation, a hallmark of cachexia, and negatively impact cancer prognosis (35). These inflammatory markers may influence tumor behavior, treatment response, and overall survival.

In contrast, SFA, while still important for energy storage and metabolic regulation, is less metabolically active and typically has fewer direct associations with inflammation and systemic metabolic changes associated with cachexia (36). This could explain why it did not emerge as a significant prognostic factor in our study.

A previous study on gastric cancer have shown that SFA may be a prognostic indicator. This difference could stem from the distinct metabolic and inflammatory profiles of gastric cancer. Gastric cancer patients may exhibit a different pattern of fat distribution compared to colon cancer patients (37). In gastric cancer, SFA may have a more pronounced relationship with cachexia due to its interactions with other specific metabolic pathways and tumor behavior (23), which might not be as prominent in colon cancer. These differences underline the importance of considering cancer-specific contexts when interpreting the role of adipose tissue in prognosis.

Furthermore, the radiological assessment of VFA and SFA provides additional insights into their prognostic value. VFA reduction may be associated with specific imaging features such as increased intraperitoneal tumor burden, tumor infiltration, or organ involvement. These changes could indicate more advanced disease progression and greater systemic metabolic impact, reinforcing the prognostic significance of VFA. While our study did not focus on detailed radiological correlations, the potential link between visceral fat loss and tumor burden suggests that incorporating imaging-based assessments could further enhance prognostic stratification in clinical practice. Future studies utilizing radiomics and texture analysis may provide a more comprehensive understanding of how body composition changes reflect disease severity and treatment response in colon cancer patients with cachexia.

The findings of this study highlight the significance of assessing and monitoring VFA in clinical practice. Future research should further investigate strategies to improve the prognosis of colon cancer patients with cachexia, including management of VFA loss and its potential impact on therapeutic interventions and quality of life enhancement.

This study is inherently limited by its retrospective design, including potential selection bias and detection bias. Additionally, it lacks critical information, such as disease-free survival (DFS). We did not specifically validate the cachexia definition based on weight loss and BMI metrics across other patient subgroups or in populations with atypical presentations of cachexia. Moreover, we did not investigate whether inflammatory markers might influence patient survival, which could provide valuable insights into the complex mechanisms of cachexia. Notably, our study was conducted exclusively among a Chinese patient population, characterized by a lower average BMI compared to the global average. This characteristic further highlights the potential limitations in the generalizability of our findings. To address these limitations, multicentric international standardized studies and long-term follow-up are needed to assess local recurrence, long-term survival outcomes in diverse populations.

In conclusion, preoperative VFA, but not SFA was a useful prognostic factor for long-term outcomes in stage I-III colon cancer with cachexia. More attention should be paid to VFA in colon cancer patient with cachexia.

## Data Availability

The original contributions presented in the study are included in the article/Supplementary material, further inquiries can be directed to the corresponding author.

## Footnotes

^1^ http://www.Rproject.org/

## Supplementary material

Supplementary Figure S1 The flow chart of patients enrolled in the study.

Supplementary Figure S2 Kaplan-Meier survival curve for colon cancer patients stratified by cachexia (cachexia vs. non-cachexia).
