# Supplementary figures and images for: Prognostic impact of visceral and subcutaneous fat area in stage I-III colon cancer patients with cachexia: a population-based multicenter study
Source: Front Nutr. 2025 Mar 3;12:1538285. doi: 10.3389/fnut.2025.1538285 (PMC11911208; doi:10.3389/fnut.2025.1538285)

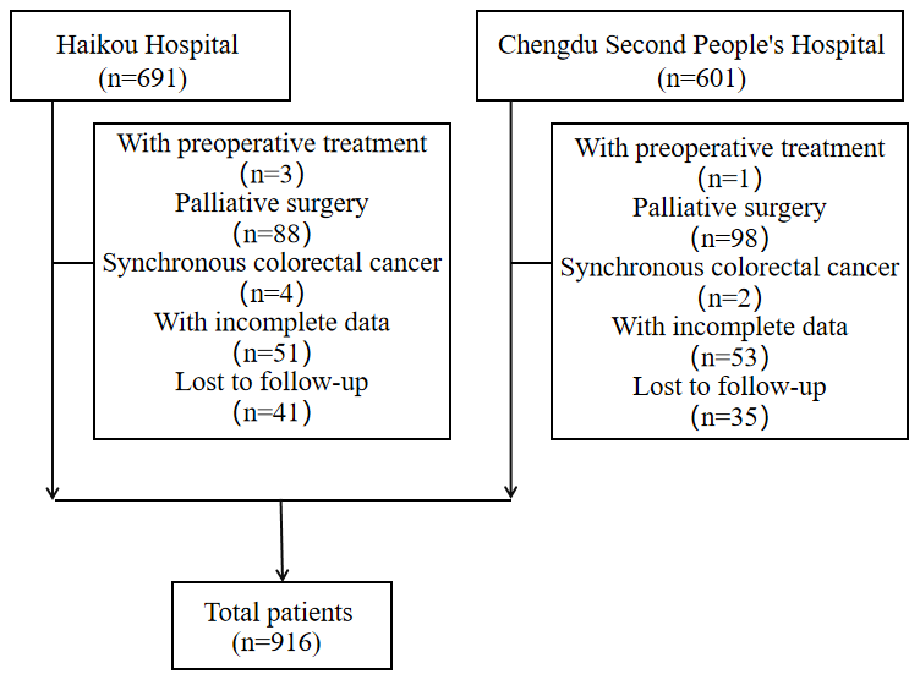

Supplement: Supplementary Figure S1 — The flow chart of patients enrolled in the study. [file Figure_1.TIF]

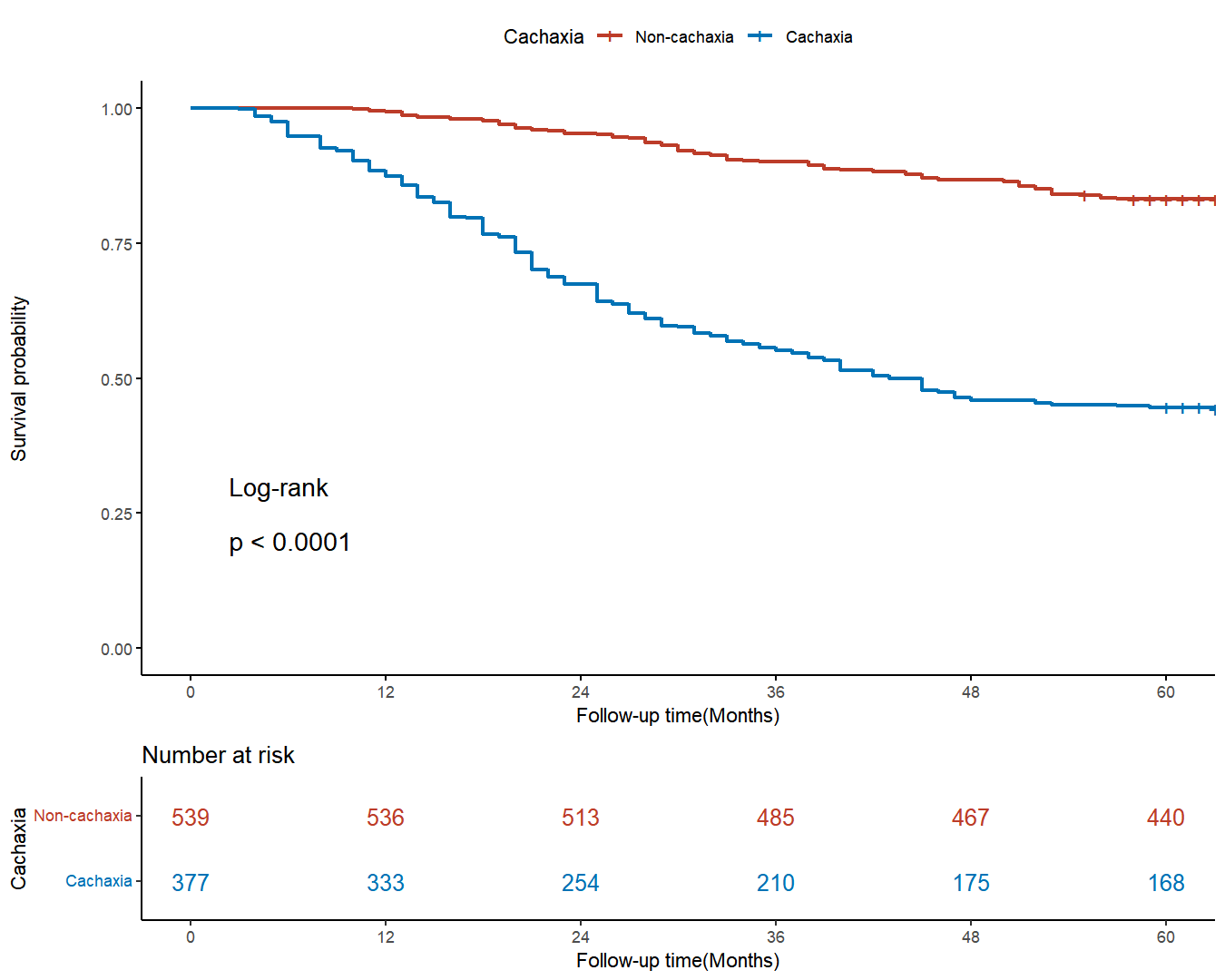

Supplement: Supplementary Figure S2 — Kaplan-Meier survival curve for colon cancer patients stratified by cachexia (cachexia vs. non-cachexia). [file Figure_2.TIFF]
