# Supplementary material for: Prognostic impact of visceral and subcutaneous fat area in stage I-III colon cancer patients with cachexia: a population-based multicenter study
Source: Front Nutr. 2025 Mar 3;12:1538285. doi: 10.3389/fnut.2025.1538285 (PMC11911208; doi:10.3389/fnut.2025.1538285)
Supplement: Supplementary file 3 [file Table_1.DOCX]

**Table S1. Baseline characteristics of the total patients**

| Clinicopathologic characteristics | Total (N=916) |
| --- | --- |
| Sex |  |
| Male | 540 (59.0) |
| Female | 376 (41.0) |
| Age (year)* | 61.0 (51.0, 69.0) |
| ASA score |  |
| 1-2 | 590 (64.4) |
| 3-4 | 326 (35.6) |
| Hypertension |  |
| No | 763 (83.3) |
| Yes | 153 (16.7) |
| Heart disease |  |
| No | 889 (97.1) |
| Yes | 27 (2.9) |
| Chronic pulmonary disease |  |
| No | 851 (92.9) |
| Yes | 65 (6.1) |
| Diabetes mellitus |  |
| No | 825 (90.1) |
| Yes | 91 (9.9) |
| CEA level (ng/ml) * | 2.6 (1.5, 6.1) |
| Albumin (g/L)* | 38.3 (35.8, 40.9) |
| BMI (kg/m^2^) * | 22.4 (20.5, 24.3) |
| VFA (cm^2^ ) * | 83.1 (52.8, 114.8) |
| SFA (cm^2^ ) * | 103.4 (72.9, 152.1) |
| Surgical approach |  |
| Laparoscopy | 565 (61.7) |
| Laparotomy | 351 (38.3) |
| Pathologic tumor category |  |
| T1-2 | 92 (10.0) |
| T3-4 | 824 (90.0) |
| Pathologic node category |  |
| N0 | 567 (61.9) |
| N1-2 | 349 (38.1) |
| AJCC 8th staging |  |
| I | 71 (7.8) |
| II | 496 (54.1) |
| III | 349 (38.1) |
| Perineural invasion |  |
| No | 770 (84.1) |
| Yes | 121 (13.2) |
| Not reported | 25 (2.7) |
| Lympho-vascular invasion |  |
| No | 727 (79.4) |
| Yes | 177 (19.3) |
| Not reported | 12 (1.3) |
| Tumor differentiation |  |
| Well | 260 (28.4) |
| Moderate | 543 (59.3) |
| Poor | 113 (12.3) |
| Adjuvant chemotherapy |  |
| No | 514 (56.1) |
| Yes | 402 (43.9) |

ASA, American Society of Anesthesiology; BMI, body-mass index; VFA, visceral fat area; SFA, subcutaneous fat area.
